# Auditory Cortex Processes Variation in Our Own Speech

**DOI:** 10.1371/journal.pone.0082925

**Published:** 2013-12-13

**Authors:** Kevin R. Sitek, Daniel H. Mathalon, Brian J. Roach, John F. Houde, Caroline A. Niziolek, Judith M. Ford

**Affiliations:** 1 Mental Health Service, San Francisco Veterans Affairs Medical Center, San Francisco, California, United States of America; 2 Department of Psychiatry, University of California San Francisco, San Francisco, California, United States of America; 3 Department of Otolaryngology, University of California San Francisco, San Francisco, California, United States of America; VU University Amsterdam, The Netherlands

## Abstract

As we talk, we unconsciously adjust our speech to ensure it sounds the way we intend it to sound. However, because speech production involves complex motor planning and execution, no two utterances of the same sound will be exactly the same. Here, we show that auditory cortex is sensitive to natural variations in self-produced speech from utterance to utterance. We recorded event-related potentials (ERPs) from ninety-nine subjects while they uttered “ah” and while they listened to those speech sounds played back. Subjects' utterances were sorted based on their formant deviations from the previous utterance. Typically, the N1 ERP component is suppressed during talking compared to listening. By comparing ERPs to the least and most variable utterances, we found that N1 was less suppressed to utterances that differed greatly from their preceding neighbors. In contrast, an utterance's difference from the median formant values did not affect N1. Trial-to-trial pitch (*f0*) deviation and pitch difference from the median similarly did not affect N1. We discuss mechanisms that may underlie the change in N1 suppression resulting from trial-to-trial formant change. Deviant utterances require additional auditory cortical processing, suggesting that speaking-induced suppression mechanisms are optimally tuned for a specific production.

## Introduction

Speech is a complex social and motor act, and as we speak, we unconsciously adjust our speech to more closely match the external expectations of our peers as well as our own internal expectations of what our speech should sound like [1]–[3]_ENREF_1. Socially, speakers unconsciously alter their pronunciation to better mirror the speakers around them [2], [3]. Internally, when our speech is artificially perturbed while we talk, we unconsciously alter our voice to match the sounds we intended to produce [1]. It follows, then, that speech perception influences speech production at the level of the speech sound. The brain may accomplish this iterative sensory-motor-sensory looping process through a largely unconscious forward model mechanism that allows for the anticipation of the vocal output, the assessment of the match between the expected and observed output, and the adjustment to the motor plan to correct the movement in the moment [4], [5].

A growing body of research explores the role of auditory feedback in speech production by altering what subjects hear as they speak. Over a century ago, it was noticed that when background noise is increased, speakers react by increasing their speaking volume, a phenomenon known as the Lombard Effect [6], [7]. More recently, on-line manipulation of *f*0 (fundamental frequency) in one direction has been shown to cause speakers to alter their fundamental frequency in the opposite direction [8]. Such frequency compensation is even greater when vocal folds are anesthetized, suggesting a crucial role of both somatosensory and psychoacoustic feedback in speech control [9]. Direct cortical recordings during an *f0* manipulation speaking and listening experiment are beginning to help us understand the role of feedback in speech control [10]. Compensation for altered feedback is also seen with on-line formant manipulation, where subjects unknowingly change their vowel production in the opposite direction of the altered feedback, with such adaptation persisting across trials even in the absence of altered feedback [1], [11]–[14]. A mismatch between expected and actual auditory feedback results in increased BOLD activity in the superior temporal cortex [15], [16]. Error correction to modulated feedback is also present in birdsong [17]. Thus, auditory feedback mechanisms may be universal to vocally communicative species and not limited specifically to the human capacity for speech.

The working parts of the forward model system that likely underlie compensation for modulated feedback have been described alternately as “efference copy” and “corollary discharge.” These neural signals allow us to distinguish between stimuli resulting from our own actions and sensations coming from external sources, a distinction that is crucial to survival in a potentially dangerous or hostile environment [18]–[20]. Additionally, they may allow us to implement some measure of feedback control to ensure that our movements are successful [11], [12]. Although corollary discharge and efference copy are often used interchangeably, it is helpful to distinguish between them. During motor planning, an “efference copy” of the motor action is sent to the sensory cortex, where it arises as a “corollary discharge” of the expected sensory consequences of the motor action. When there is an exact match between the actual event and the expected event, sensory responsiveness is suppressed [21], [22]. The current study focuses on the degree of match between the current speech sound and the immediately previous one during talking.

The efference copy and corollary discharge mechanisms have been studied during vocalization using invasive techniques across the animal kingdom, from crickets and bats to marmoset monkeys and humans [18], [23]–[29]. In all cases, auditory responsiveness is suppressed when the animal is vocalizing. Similar findings have been reported in humans using non-invasive techniques such as scalp recorded electroencephalography (EEG) and magnetoencephalography (MEG), which measure electrical and magnetic fields, respectively. In these studies, the N1 of the EEG-based event-related potential (ERP), or the M100 of the MEG-based response, reveals increased activity in the auditory areas of the cerebral cortex during speaking, but the level of activity is lower than that observed when the same speech sound is recorded and played back to the speaker, who passively listens to the sequence of sounds. This speaking-induced suppression [30] is interpreted as reflecting the action of the efference copy and corollary discharge systems on auditory cortical processing [11], [21], [31], [32].

The auditory N1 is generated in primary and secondary auditory cortex [33], and its amplitude increases with sound intensity and relevance. As such, it might be considered an index of the amount of resources dedicated to processing a stimulus. Suppression of N1 during talking reflects more cost-effective processing of expected sounds; when there is not a match between the expected and the actual sounds during talking, processing costs are high.

In our study, subjects repeated the vowel sound “ah” every few seconds and later heard their speech played back to them, all while EEG activity was recorded. This setup allows us to compare the N1 elicited by speech sounds between talking and playback conditions, as well as between utterances with high and low pair-wise variability. Our paradigm is optimal for investigating speech-related cortical activity for a number of reasons. First, producing the sound “ah” in isolation instead of a more complex utterance minimizes muscle activity, which would otherwise introduce noise into the EEG recordings. Second, by using a common speech sound that does not convey any semantic information on its own, we can investigate the mechanisms underlying speech production without needing to account for potentially confounding linguistic or cognitive processing. Third, because subjects in our study received minimal instruction or prompting and were not instructed to produce utterances consistently or at specific times, any variability in speech utterances arises from natural speech variability.

Using the N1 component of the ERP, we asked, what is the auditory cortical response to the natural variability of speech sounds during talking? Specifically, does an utterance that is *inconsistent with* (“Far” from) the previous utterance elicit a larger N1 than an utterance that is *consistent with* (“Near” to) its immediately preceding neighbor? Additionally, is the brain sensitive to this variation while passively listening to a recording of that speech? Lastly, can we verify that speech output variability is sequentially dependent?

## Results

### Task and behavioral data

Subjects were instructed to say “ah” at their own pace over the course of 187 seconds [34]–[36]. The number of utterances varied from 44 to 179 (mean = 90.5; S.D. = 24.4). The average interval between two “ah” onsets was about 2 seconds (mean = 1984.0 ms; S.D. = 556.7). The mean utterance duration was 262.4 ms (S.D. = 86.8).

### N1 amplitude

**Figure 1:**
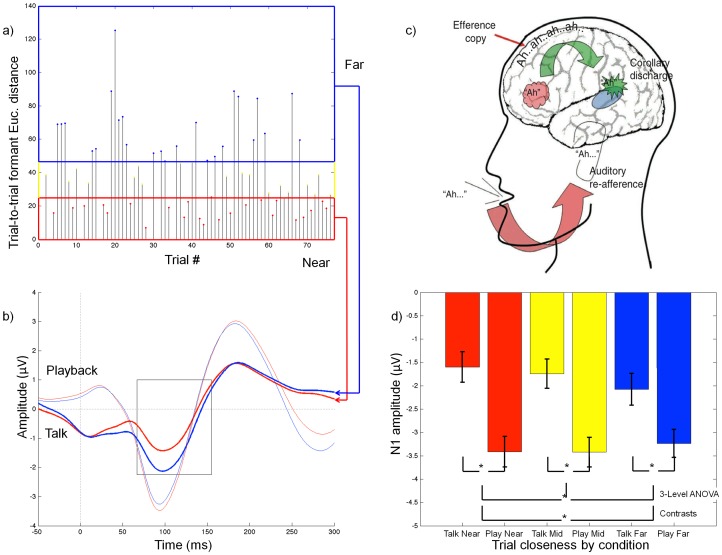
Relationship between trial-to-trial formant change and N1 suppression. Examples of trial sorting for one subject, a forward model, and the average event-related potentials (ERPs) of similar and dissimilar trials across all subjects. (a) We estimated each “ah” utterance's first two formant frequencies to find the Euclidean distance between each “ah” and its preceding neighbor. We grouped these trials into thirds: Near, Mid, and Far (referring to their formant similarity to the preceding utterance) and trimmed-mean averaged each grouping together. (b) Grand average ERPs from 99 subjects, recorded from the midline frontal site (Fz), for Talk and Playback conditions for Near and Far trials. Data points with significant Talk Near versus Far differences boxed and include the N1 ERP component (paired t-test, two-tailed, *p*<0.05). (c) Proposed framework for our findings. Premotor cortex sends an efference copy of a planned action to auditory cortex, where a corollary discharge is formed that represents the expected sensory consequences of the planned speech act. The actual percept is then compared to the predicted percept. Mismatch between predicted and actual percepts may be responsible for reduced suppression during Talk (from Mathalon et al. 2008)[35]. (d) Average N1 amplitude across all subjects and fifteen frontal-central electrodes with standard error bars. Talk versus Playback N1 effect at all trial groupings represents N1 suppression (*p* = .001). We found an overall trial Consistency effect on N1 suppression (*p* = .048) as well as a greater Near versus Far effect on SIS (*p* = .014), showing decreased N1 suppression when an utterance varies highly from its previous neighbor.

We recorded the ERP to speech onset during talking and playback of the recorded speech train, and we measured the N1 component to speech onset during Talk and Playback conditions. We confirmed the N1 suppression during Talk compared to Playback in a repeated measures analysis of variance (*F*_1, 98_ = 12.36, *p* = .001; Table 1). This can be seen in Figure 1b where we overlay the ERPs during Talk and Playback. For each subject, speech trials were sorted into tertiles (Near, Mid, and Far) based on trial-to-trial Consistency, computed as each trial's two-formant Euclidean distance from the previous trial (Fig. 1a); the ERPs of the trials in each tertile were averaged together. This trial-to-trial Consistency interacted with Condition to have a significant effect on N1 suppression (*F*_2, 196_ = 3.09, *p* = .048; Fig. 1d). In Figure 1b, Near and Far Talk ERPs are significantly different from each other at all data points within the box (all ps<.05). When contrasting the three levels of Consistency (Near, Middle, and Far), trials that were Near their preceding neighbors had significantly more N1 suppression than trials that were Far from their neighbors (*F*_1, 98_ = 6.23, *p* = .014). The contrasts between Near and Mid (*F*_1, 98_ = .230, *p* = .632) and Mid and Far (*F*_1, 98_ = 3.222, *p* = .076) were not significant.

**Table 1:** ANOVA results for the N1 ERP, with trials binned by trial-to-trial (lag 1) formant Euclidean distance.

Measure	df	F	sig.
Anterior-Posterior (AP)	1.249	49.775	<0.001
Laterality (Lat)	2.627	19.481	<0.001
Condition (Cond)	1	12.359	0.001
Cond * Lat	2.812	4.789	0.004
Cond * Consistency	1.971	3.091	0.048
Near versus Far contrast	1	6.23	0.014
Cond * AP	1.262	3.025	0.075
Cond * Lat * Consistency * AP	10.995	1.088	0.367
Cond * Consistency * AP	2.523	0.967	0.398
Cond * Lat * Consistency	4.517	0.888	0.481
Consistency	1.952	0.61	0.54

*AP* (anterior-posterior) and *Lat* (laterality) reflect electrode location and include frontal, frontal-central, and central AP electrode bands from five lateral bands including and around the midline. We found that *Cond* (Condition: Talk and Playback) interacted with *Consistency* (an utterance's formant similarity to the previous utterance: Near, Mid, and Far) to have a significant effect on N1 size. Summary of repeated measures analysis of variance for the N1 event-related potential component, using Greenhouse-Geisser correction for sphericity when appropriate.

### Talk versus Playback condition

It is important to note that N1 suppression differences were driven by the Talk condition. In separate ANOVAs, Consistency was significant during the Talk condition (*F*_2, 196_ = 3.26, *p* = .040), especially when contrasting responses to Near and Far utterances (*F*_1, 98_ = 6.37, *p* = .013). There were no significant changes in N1 in the Playback condition for overall Consistency (*F*_2,196_ = .594, *p* = .551) nor for Near versus Far contrasts (*F*_1, 98_ = .836, *p* = .363).

### Distance from median utterance

In another analysis, instead of sorting trials by comparing the formant values of a trial to those of its immediately preceding neighbor, we compared the trial's formant values to the median formants of all trials. Thus, a trial binned into the Far tertile has a large Euclidean distance from the median F1 and median F2 values across all trials, regardless of whether it is similar or different from its immediately preceding neighbor. With this distance-from-median analysis, we found no Consistency by Condition interaction (*F*_2,196_ = .336, *p* = .693).

### Comparison of different lags

**Figure 2:**
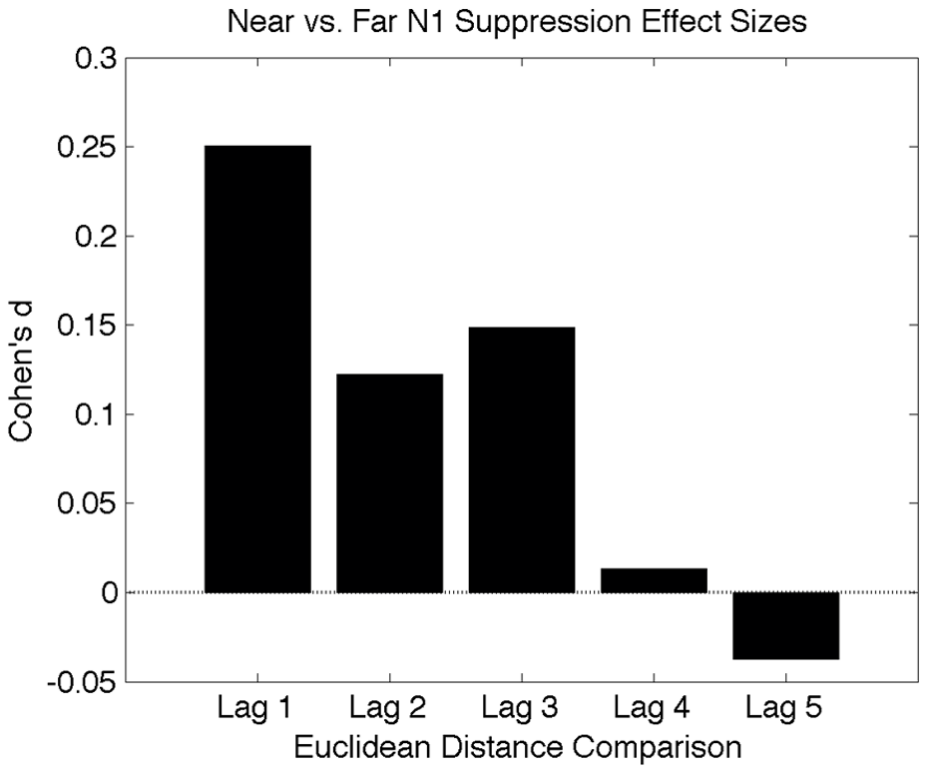
Cohen's d effect sizes across lags. Cohen's d effect sizes of Talk minus Playback N1 event-related potential (ERP) suppression for Near versus Far trials when compared in formant space to the most recent trial (Lag 1), the second-most recent trial (Lag 2), etc., through Lag 5. Although only Lag 1 Near vs. Far formant change had a significant effect on N1 suppression, there is a linear relationship between lag and Near vs. Far N1 suppression effect size (*p* = .023).

To test for a relationship between the current utterance and other recent utterances, we compared the Euclidean distance between an utterance and the most recent utterance (lag1), the second most recent utterance (lag2), and so on through lag5 (Figure 2). As noted above, sorting trials by lag1 (current vs. most recent utterance) formant change reveals a significant Consistency x Condition interaction. No other lags had significant interactions (*F_2,196_*<1.25, *p*>.2). We added the factor “Lag” to our statistical model and found an interaction effect with Condition x Consistency x Lag when contrasted linearly (*F_1,98_* = 5.323, *p* = .023). There were no significant pairwise differences between individual lags beyond lag1 vs. lag5 (*F_1,98_* = 5.070, *p* = .027).

### Pitch (f0)

We investigated whether pitch, or fundamental frequency (*f0*), affected N1 suppression by sorting trials based on their *f0* deviation from the previous utterance as well as by their deviation from the median *f0* value. Neither approach yielded significant relationships (*F*<1.5, *p*>.23).

### Intensity

Additionally, we sorted trials by their intensity by measuring the recorded utterance's root mean square (RMS) amplitude. Trials were sorted into tertiles based on their RMS values, giving us “quiet,” “medium,” and “loud” trial groupings. We found no Intensity by Condition interaction on N1 suppression (*F*_2,196_ = 2.71, *p* = .072). Although trending toward significance, the effects were opposite of these seen for sorting based on formant differences: in separate Condition analyses, Intensity had a non-significant effect on N1 during Talk (*F*_2,196_ = 1.67, *p* = .190) but a significant effect during Playback (*F*_2,196_ = 3.23, *p* = .042) such that louder sounds elicited larger N1 components. See below for Talk vs. Playback discussion.

We separately sorted trials based on trial-to-trial change in RMS amplitude and found a significant Condition by trial-to-trial Intensity change interaction (*F*_2,196_ = 3.96, *p* = .021) driven by decreased N1 suppression for second tertile trials. However, we found no linear Intensity change by Condition contrast (*F*_1,98_ = 1.029, *p* = .313). In follow-up analyses, neither Talk condition (*F*_2,196_ = 2.84, *p* = .061) nor Playback condition (*F*_2,196_ = 1.67, *p* = .191) had a significant trial-to-trial Intensity effect, and neither had a linear contrast across tertiles (*F*<1.1, *p*>.30).

### Stimulus onset asynchrony (SOA)

Similarly, we analyzed the trials based on how quickly they followed the previous trial, grouping trials into “short,” “medium,” and “long” SOA tertiles. We found no SOA by Condition interaction (*F*_2,196_ = .09, *p* = .914). We separately sorted trials based on trial-to-trial change in SOA and found no Condition by trial-to-trial SOA change interaction (*F*_2,196_ = 2.33, *p* = .10) nor linear trial-to-trial SOA by Condition contrast (*F*_1,98_ = 1.379, *p* = .243). Talk condition did have a significant effect of trial-to-trial SOA (*F*_2,196_ = 3.415, *p* = .035) driven by a small second tertile N1. There was no linear contrast (*F*_1,98_ = 1.257, *p* = .265) for Talk condition trial-to-trial SOA-sorted trials, and neither a Playback condition trial-to-trial SOA effect nor linear contrast (*F*<.5, *p*>.5).

### Speech autocorrelation

To examine how speech utterances influence their neighbors, we investigated the relationship between the formants of sequential speech trials by finding the autocorrelation of formant Euclidean length change across trials. (See Methods for more details.) In an analysis of variance across eleven lags, the negative lag-one correlation (*r* = −.47, Fisher-transformed) differed greatly from all other lags (*F*>240, *p*<.001). Correlations at other lags remained between +/−.05. However, across subjects, lag-one autocorrelation scores were uncorrelated with Near-Far N1 suppression change and overall N1 suppression.

### ERP-behavioral correlations

Age correlated marginally with overall Talk-Play suppression at midline electrodes (Fz: *r* = .195, *p* = .053; Cz: *r* = .203, *p* = .044), with N1 suppression increasingly slightly with age. However, there was no relationship between age and Near-Far ERP differences (−.03 <*r*<.08, *p*>.45).

## Discussion

Speakers suppress auditory processing of their own speech more when the speech sound is similar to the most recent production. Our findings suggest that the auditory cortex is sensitive to slight variations in speech during talking. The subjects in this study had no instructions to produce consistent speech sounds, suggesting that the brain's sensitivity to deviations in speech production is invoked automatically without strategic, top-down control. The larger N1 to deviations from the previous sound suggests an additional allocation of auditory cortical resources, an energetically expensive process. Importantly, it was trial-to-trial change, not deviation from the median of all utterances, which resulted in a larger N1 in this task. This suggests that auditory cortex compares the current utterance to the previous utterance during repeated productions of the same utterance.

Why is an utterance's deviation from the previous trial the driver of the N1 effect, and not the deviation from the median utterance? Niziolek, Nagarajan, and Houde suggest that proximity to the centroid of a vowel's range of production alters cortical activity 100 ms after vowel onset [37]. However, there are a few key differences between the two studies. First, while Niziolek et al.'s study asked participants to say real English words with various vowels (“eat,” “Ed,” and “add”), our experiment only prompted participants to produce a particular-sounding utterance, “ah” (/a/). /a/ is a common speech sound in American English, but it conveys no meaning on its own–it is only in combination with other phonemes that this speech sound can represent a concept. Additionally, Niziolek et al. compared a trial's formants to the median formants using the average of the first 50 ms; here, we averaged the formant values from the entire utterance. Finally, and perhaps most importantly, speakers in our study were able to directly compare their current utterance with their previous utterance because all of the utterances were the same phoneme and thus had the same motor target. Niziolek et al.'s task prompted speakers to say a variety of utterances quasi-randomly, so this repetitive motor planning model was not available to the speakers. Thus, the different findings of these two seemingly similar studies actually represent different processes: (1) the comparison of a spoken word to cognitively stored stereotypes of its elements; versus (2) the comparison of one of a series of repeated spoken utterances to the auditory consequences of the previous effort to perform the same action.

It is critically important to note that the difference in N1 suppression for the different degrees of deviance (Near vs. Far) was due to the effects during Talk, not Playback. If we had seen similar changes in the Playback condition, then we could attribute the Near versus Far N1 differences to purely auditory mechanisms, unrelated to the speech motor plan. Indeed, there is a large literature showing enhanced cortical responsiveness to deviant sounds during listening. Deviant sounds can elicit larger N1, mismatch negativity (MMN), and P3a sensory-specific ERP components compared to non-deviant or standard sounds [38]–[40]. In our study, these components were not elicited by the sounds that differed from the previous sound during passive listening. Because the Talk condition was significantly affected but not the Playback condition, our findings reflect an active speech-related feedback mechanism and not more basic auditory perception processes.

In fact, the forward model mechanism at work when looking at trial-to-trial deviation seems to overpower more general auditory processing of self-produced speech. When sorting trials by intensity (RMS amplitude), Talk condition N1 does not vary, while Playback condition N1 is larger for louder utterances. Since Talk condition N1 does not change with intensity, the predicted sensory feedback must include information about the planned intensity of the impending self-produced sound.

It is also worth noting that trial-to-trial formant deviation was the only speech measure to evoke differences in N1 suppression. Differences in utterance intensity, time between utterances, and pitch (both trial-to-trial change and distance from the median) were not correlated with changes in N1 amplitude. Although we gave subjects very few instructions, the main requirement was to produce the “ah” vowel sound. In English, vowels do not contrast in pitch, loudness, or any feature other than formants, so our task primarily demanded formant control. However, we also instructed subjects to produce utterances about every two seconds and at a comfortable speaking volume around 80 dB SPL, yet we found no affect of SOA or loudness on the N1. Thus, because of its importance to vowel differentiation, we believe that formant control is most critical in utterance production, even when the utterance is a simple phoneme carrying no linguistic information.

While we found a strong effect of trial-to-trial formant variability on the N1 component, the purpose of this trial-to-trial comparison is not clear. There are multiple possible explanations for our findings, each of which assumes a different goal and underlying mechanism. For example, if the goal is to minimize production errors and constantly improve utterance production so as to “home in” on a target production, then models of speech motor control will help us understand the mechanisms at play. However, N1 variability based on trial-to-trial formant change could represent the activity of other goals such as sensory prediction coding and consistency monitoring, attention, or intentional variability. Thus, the findings highlighted above may come from any of a variety of cortical processing mechanisms.

Motor control models attempt to describe how people learn from previous attempts at a movement in order to better perform that action. Even with a well-defined motor goal and in the absence of perturbed sensory feedback, noise in the execution of the motor command causes actual movements to vary from trial to trial. According to the planned aim point correction (PAPC) model, previous motor signals are referenced to plan the next one. Crucially, “corrections are made relative to the previous movement's planned aim point,” so both planning and uncontrollable motor noise influence the next movement's aim point [41]. Previous speech research found a negative correlation between sequential utterances [42], hinting that repetitive speech may function as a “memoryless” Markov chain where the outcomes of speech acts beyond the most recent utterance are unavailable to the speech planning mechanism. Importantly, Purcell and Munhall found a correlation between utterance-to-utterance changes and compensation for altered speech feedback such that subjects with the greatest compensation had the most negative sequential utterance difference correlation. Other motor control models focus on speech explicitly. For example, in the state feedback control (SFC) framework, within-utterance feedback compares expected auditory consequences to the actual auditory percepts [4], [5]. The DIVA (Directions Into Velocities of Articulators) model of speech production also accounts for within-utterance corrections, where experimenter-altered auditory feedback encourages a compensatory change in production while unaltered somatosensory feedback resists any changes [5], [13]. However, SFC only accounts for within-utterance corrections, while DIVA updates its motor program after each utterance in order to converge on the target, but only during the learning phase.

The effect of trial-to-trial formant variation on the cortical N1 component in our study suggests that a PAPC-like framework could underlie speech motor control. In the PAPC model, feedback from the actual endpoint of the previous movement is compared to the task target, which stays constant across all trials. The next motor planned aim point corrects for the error of the previous trial–that is, the mismatch between the target and actual endpoint that results from a combination of planning error and unknowable random noise. Thus, each movement takes into account the success or failure of the previous movement to match the target and updates its plan accordingly. Here, our findings suggest a comparison between the current utterance and the most recent utterance, where large differences between sequential utterances are correlated with increased auditory cortical activation. As previously described, a speaker's utterance tended to move in the opposite direction as the last utterance in terms of the first two formants, although we did not find any correlations between the strength of these sequential changes and the N1 [42]. A key difference between our findings and the PAPC model is that in our study, the comparison between sequential utterances is paramount, whereas the comparison between the previous movement's outcome and the task target is central to PAPC. Perhaps the (relative lack of) constraints of our task encouraged subjects to strive for trial-to-trial consistency instead of matching a task target. Additionally, although comparison to utterances beyond the most recent was statistically unrelated to N1 suppression, there was a significant linear decrease across these lags, which does not fit with the PAPC model and warrants further investigation. Whatever the reason, our findings are inconsistent with the current PAPC model but could fit a model that corrects for the error in a movement toward a different (or varying) task target.

We can also approach our findings from a sensory prediction model instead of an error correction model. The predictive coding framework of brain function posits that the goal of perception is to minimize sensory prediction errors [43]. An unexpected sound updates the auditory stimulus prediction, a process likely involving the mismatch negativity (MMN) ERP. The MMN was first evoked by replacing a usual “standard” stimulus with an unexpected “deviant” one but has since been reported in more complex environments [44], [45]. Predictive coding can also be applied to predictions of sensory consequences of one's own movements where the predictions can be sampled by motor processes to monitor performance and update the motor plan. Our findings in this study may arise from the mismatch between two adjacent utterances. If the sensory system expects an utterance to sound like the previous production, an utterance that varies from the previous production may be processed as a deviant, as the sensory consequence of the second production does not match the predicted auditory sensation. A poor sensory prediction could arise from an error in production that surprises the sensory system, but it could also stem from decreased resources being allocated to sensory prediction, as our study involved the possibly boring task of repeating of the same vowel sound for three minutes. Of course, any predictive coding mechanisms must explain the talking-specific effect, as the same utterances elicited no significant changes in N1 when played back to the speaker.

Alternatively, instead of being a production “mistake” that must be corrected or perception-production mismatch, variability may instead be intentional, at least within certain bounds. Purposeful variation within a speech category (like the phoneme /a/) could be used to test the range of acceptable productions of the same speech sound. In this case, the N1 would represent a variability-monitoring mechanism that tracks excursions away from the most recently produced utterance. Indeed, studies of auditory feedback in songbirds suggest that motor noise and trial-to-trial variability can be used in this manner, especially when learning a new vocalization [17].

As just outlined, there are a number of mechanisms that could underlie the effect of trial-to-trial formant deviation on speaking-induced suppression. Our task is designed to compare cortical responses during speech production to passive listening of one's own speech [34]. Experimental simplicity is key to this investigation, which has previously uncovered cortical differences between healthy speakers, patients with schizophrenia, and young participants at high risk for developing a psychotic disorder [35], [36]. Unfortunately, this simplicity makes it difficult for our study to solve mechanistic questions of underlying processes. However, our present findings are unique in the field of speech research, and they provide novel insight into brain functioning during speech. Any explanation for our findings will have to account for the N1 effect of small formant changes during speech but not during playback of one's own speech.

Our findings are consistent with the single unit findings of Eliades and Wang (2005), who reported that variability of neural response is related to variability of vocalization frequency and energy (which were highly correlated with each other) [25]. However, our study extends their study in important ways. First, our study showed sequential effects of subtle deviance by showing that auditory cortex is sensitive to deviations in vocalization from the immediately preceding one. Second, we showed this in humans using non-invasive methods.

Speech is a complex motor action that requires constant on-line feedback to ensure that the correct vocalization is produced. Based on our findings, we now know that the auditory cortex monitors repeated speech output at least in part by comparing an utterance to its most recent neighbor. Utterances that vary greatly from their neighbors produce a larger N1 ERP component, signifying increased stimulus processing. There are no analogous N1 differences when subjects hear their own utterances played back to them, suggesting that the N1 is affected by speaking-specific feedback monitoring mechanisms. Further research will elucidate the specific cortical mechanisms used to ensure phonemic consistency.

## Methods

We have previously described the general acquisition procedures and ERP processing stream [34], [36]. The appropriateness of the task in investigating elements of the forward model is discussed in Mathalon & Ford [35]. Below are the most relevant features of the analysis, as well as analysis methods specific to this paper.

The UCSF institutional review board and San Francisco VA Research Office approved this study.

### Participants

99 subjects (mean age  = 27.45, min = 12.8, max = 62.1, SD = 11.005) were recruited by online advertisements, flyers, and word-of-mouth. All subjects had normal hearing between 250 Hz and 4000 Hz as determined by a test of pure-tone auditory threshold. Adult subjects provided written consent of being informed of study procedures. For subjects under 18 years of age, parents provided written informed consent and minors provided written assent.

### Procedure

Subjects first produced “ah” utterances (“Talk” condition) and later heard their utterances played back to them (“Playback” condition).

In the Talk condition, subjects were asked to say the phoneme “ah” every 1–2 seconds for 187 seconds. Each participant's speech was recorded with a microphone placed near the mouth and sent to the stimulus presentation computer, which was monitored in real time through the subject's Etymotic ER3-A ear inserts.

Next, in the Playback condition, subjects were instructed to listen as their recorded speech was played back through their earphones.

### Acoustic calibration and standard stimulus generation

Before beginning EEG and speech recording, the researchers observed the participant producing practice utterances and guided the participant to speak a typical speaking intensity. Using a hand-held sound level meter held ∼5 cm from the participant's mouth, the researchers ensured that the “ah” vocalizations were between 75–85 dB SPL. Talk and Playback conditions outputted the speaker's utterances at the same intensity, which was calibrated with a 1000 Hz tone generated by a Quest QC calibrator played through the earphones.

Each participant's Talk condition speech was digitized at 44.1 kHz and saved as a .wav file, which was played back during the Playback condition. Additionally, the speech file was processed with an automatic speech onset-finding algorithm (see Ford et al. [34] for further details) [34]. The onsets were saved as triggers, which were later used for ERP analysis (see below).

### Data acquisition and pre-processing

See Ford et al. (2010) and Perez et al. (2011) for details about EEG collection and initial processing [34], [36].

Epochs were based on the automated vocalization onset-finding algorithm, which were additionally fine-tuned manually. Further processing occurred after epochs were binned based on speech features (see next section) but will be described here. The processed data, after binning, underwent trimmed-mean averaging. This approach eliminates the highest 25% and lowest 25% of values at each time point, averaging together the remaining 50% of values for each point into an ERP average [46].

Temporal Varimax-rotated principal component factor analysis identified noise components that did not contribute to the event-related potential. Components were excluded from back-projected ERPs if they accounted for <1% of the signal variance (∼66% remaining). ERPs were baseline-corrected relative to the ERP signal from 600 ms to 500 ms before stimulus onset. For our purposes, N1 was defined as the average of the ERP between 80–110 ms. Using static area boundaries as opposed to peak-specific boundaries allows for a more robust area measurement by ignoring the potential contribution of noise to the component peak [47].

### Speech analysis

Each subject's speech data were saved as a .wav file at full acquisition sample rate and imported into MATLAB 7.9 (MathWorks, 2009). “Ah” onset time, as described above, was picked automatically and fine-tuned manually by the experimenters. Offset was determined to be the time point when the absolute value of the “ah” time waveform decreased to below 150% of the mean of the pre-speech window (−50 ms to 0 ms).

In order to measure segmental phonetic variations in “ah” utterances, we measured the formants of each “ah” using linear predictive coding (LPC) via the open source Praat phonetic analysis software (www.fon.hum.uva.nl/praat/). LPC uses the source-and-filter model of speech production to estimate formants by capturing periodicities in the waveform created by formant resonances [48]. The program was set to find five formants (10^th^ order LPC filter) with a maximum formant frequency of 5000 Hz for males and 5500 Hz for females, corresponding to sampling frequencies of 10,000 Hz and 11,000 Hz, respectively. 50 Hz pre-emphasis doubled the signal amplitude for every doubling of the frequency above 50 Hz, compensating for decreasing intensity at higher frequencies by creating a flatter spectrum for analysis. Formants were estimated in 5 ms analysis windows, comparable to a 5 ms Hamming window; however, Praat uses a Gaussian-like window with values below 4% outside the central 5 ms window, producing an actual Gaussian window of 10 ms. As American English vowels contrast across the first two formants (F1 and F2), we used F1 and F2 to find the average value of each formant across the entire utterance.

Our first step in the estimation of the Euclidean distance between the target “ah” trials and the other “ah” trials was to convert the formant frequencies from Hertz to the logarithmic-based mel psychoacoustic measure of frequency, which is a better model of the auditory perceptual system than the linear Hertz measure. We used the equation *m* = k*log(1+(*h*/700)), where *k* is a constant (here, 2595), *h* is the frequency in Hertz, and *m* is the corresponding mel frequency [49], [50].

Next, with the mel values of F1 and F2, we were able to compute the Euclidean distance of each utterance from the previous utterance (*d_Euc_^t^*). Thus, for each subject and for each trial *t* after the first trial, *d_Euc_^t^* = sqrt((F1_t_−F1_t−1_)^2^+(F2_t_−F2_t−1_)^2^).

Finally, single trials were sorted into tertiles according to the Euclidean distance between the target “ah” and the immediately preceding “ah”. The top third of the ordered “ah” trials were most similar to the target and are termed “Near” trials. The bottom third of the “ah” trials corresponded to the least similar to the target and are termed “Far” trials. The “Middle” tertile is the third of the trials in between the “Near” and “Far” trials. Average ERPs were constructed for the Near, Middle, and Far trials that immediately followed the target “ah.”

A similar process was used to compare a trial to the median of all trials. First, the F1 median and F2 median were computed across all utterances for each subject. Next, we calculated the Euclidean distance from each trial to the median utterance. Using these Euclidean distance values, we sorted each utterance into tertiles (Near, Middle, and Far).

In addition to comparing an utterance to its most recent neighbor (lag1), we compared it to its second most recent neighbor (lag2) and so on through lag5 via formant Euclidean distance. For each lag *x*, trials were sorted into Near, Mid, and Far tertiles based on their Euclidean distance from the *x*-most recent trial.

We computed pitch (or fundamental frequency, *f0*) for each utterance with cepstral analysis, which uses the power spectrum of the logarithmic power spectrum to find a peak corresponding to pitch frequency [51]. The same trial-to-trial and distance-from-median sorting methods were used as for formants above.

### Statistical analysis

N1 amplitudes were measured from 15 frontal-central scalp sites (F3, F1, Fz, F2, F4, FC3, FC1, FCz, FC2, FC4, C3, C1, Cz, C2, C4 in the 10–20 standard system) and were subjected to a four-way repeated-measures ANOVA for the factors of Condition (Talk, Playback), Consistency (Near, Middle, and Far), and two scalp distribution factors: Anterior-Posterior (labeled F, FC, or C) and Lateral (far left labeled “3”, left “1”, midline “z”, right “2”, far right “4”).

We computed autocorrelation values for each subject in a manner similar to Purcell and Munhall (2006) but using the two-formant Euclidean length value instead of just the first formant [42]. We first found the change in Euclidean length (*l_Euc_*) from one trial to the next, where for trial *t*, *l_Euc_(t) = sqrt(F1(t)^2^+F2(t)^2^)*. We subtracted the formant Euclidean length of trial *t+1* from that of trial *t*, then subtracted the mean Euclidean length across all trials, such that for each trial, the change in Euclidean length of trial *t* to the next trial is *Δ(t) = (l_Euc_(t)−l_Euc_(t+1))−mean(l_Euc_^all^)*. The autocorrelation values are then found for time lags zero through eleven. The correlation values are then Fisher r-to-z transformed to normalize their distribution for parametric statistical tests. Because lag-zero Fisher-transformed autocorrelations are infinite, analysis of variance with simple contrasts included lags one through eleven.
